# Books and Pamphlets Received

**Published:** 1884-04-20

**Authors:** 


### *BOOKS AND PAMPHLETS RECEIVED.*

*Drugs and Medicines of North America*, a Quarterly devoted to the Historical and Scientific Discussion of the Botany, Pharmacy, Chemistry and Therapeutics of the Medicinal Plants of North America, their Constituents, Products and Sophistications. J. U. Lloyd, Commercial History, Chemistry and Pharmacy. C. G. Lloyd, Botany and Botanical History. Cincinnati: J. U. & C. G. Lloyd, 189 Elm Street, 1884.

The above work, when complete, in accordance with the design of the authors, will certainly cover a wide and important field. The writer promises to "give credit irrespective of persons or prejudices," and to "search the literature of all schools of medicine, regardless of antagonism among them," "regarding the Medical and Pharmaceutical Professions as two great bodies laboring for a common purpose."

The objects and purpose thus expressed will not be objected to by progressive men in the Profession, but will be encouraged by all who desire to see our knowledge enlarged in the Materia Medica and to have placed upon record, in compact and accessible shape, all that is known of the medicinal plants of North America. The undertaking is certainly a great one, but much confidence in its successful accomplishment has been expressed by those best acquainted with the ability of the authors and their facilities for performing the work.

*Use of Peroxide of Hydrogen in Diphtheria.* By R. J. Nunn, M.D , Savannah, Ga., Physician to the Female Department of the Savannah Hospital. New York: Printed by H. A. Vonneidshutz, 69 Pearl St. 1884.

*Medical Symbolism.*—By T. S. Sozinskey, M.D., of Philadelphia. Reprint from Medical and Surgical Reporter.

*Annual Address* delivered before the American Academy of Medicine at New York October 10th, 1883, by Henry O. Marcy, A.M , M.D., President of the Academy.

*Moral Insanity—Psychosensory Insanity.*—By C. H. Hughes, St. Louis. Reprint from Alienist and Neurologist.

*Fifth Annual Report* of the Board of Health of the City of Atlanta, for the year 1883.

*Fifth Biennial Report* of the State Board of Health of Maryland, January, 1884.

